# Do modern types of wheat have lower quality for human health?

**DOI:** 10.1111/nbu.12461

**Published:** 2020-09-22

**Authors:** P. R. Shewry, K. L. Hassall, H. Grausgruber, A. A. M Andersson, A.‐M. Lampi, V. Piironen, M. Rakszegi, J. L. Ward, A. Lovegrove

**Affiliations:** ^1^ Plant Science Department Rothamsted Research Harpenden UK; ^2^ Computational and Analytical Sciences Department Rothamsted Research Harpenden UK; ^3^ Department of Crop Sciences University of Natural Resources and Life Sciences Vienna Tulln an der Donau Austria; ^4^ Department of Molecular Sciences Swedish University of Agricultural Sciences Uppsala Sweden; ^5^ Department of Food and Nutrition University of Helsinki Helsinki Finland; ^6^ Agricultural Institute Centre for Agricultural Research Martonvásár Hungary

**Keywords:** dietary fibre, gluten, minerals, phytochemicals, vitamins, wheat

## Abstract

Wheat is the major staple food in Western Europe and an important source of energy, protein, dietary fibre, minerals, B vitamins and phytochemicals. Plant breeders have been immensely successful in increasing yields to feed the growing global population. However, concerns have been expressed that the focus on increasing yield and processing quality has resulted in reduced contents of components that contribute to human health and increases in adverse reactions. We review the evidence for this, based largely on studies in our own laboratories of sets of wheats bred and grown between the 18^th^ century and modern times. With the exception of decreased contents of mineral micronutrients, there is no clear evidence that intensive breeding has resulted in decreases in beneficial components or increases in proteins which trigger adverse responses. In fact, a recent study of historic and modern wheats from the UK showed increases in the contents of dietary fibre components and a decreased content of asparagine in white flour, indicating increased benefits for health.

## Introduction

Plant breeding has been immensely successful in increasing the yield and total production of staple crops, providing food for the growing global population (Fedoroff 2010). These increases have been particularly impressive in wheat, rice and maize, the three major cereals which are the staple foods over much of the globe. In the case of wheat, which is the staple crop in the UK and Europe, global production has increased by over three‐fold between the 1960s and the present day (see http://www.fao.org/faostat/en/#data).

The major component in the wheat grain is starch, which accounts for approximately 70% of the grain dry weight. Hence, increases in yield essentially reflect increase in starch production. About half of the wheat grown in the UK, and most of the wheat produced globally, is used for human food, particularly for making bread, other baked products, pasta and noodles. The quality for these end uses is determined mainly by the gluten proteins, and hence, selection for yield in breeding programmes is usually combined with selection for grain protein content and quality.

It has been suggested that this intensive selection may have two consequences for human nutrition and health. Firstly, that selection for high starch and gluten proteins has resulted in reduced contents of other grain components that contribute to diet and health (including non‐gluten proteins, minerals, vitamins and beneficial phytochemicals). Secondly, that increases in the content of gluten and changes in gluten protein composition may have contributed to increases in adverse reactions to the consumption of wheat‐based foods (Morris & Sands 2006).

There is clear evidence that the concentrations of most mineral micronutrients, including iron, zinc and magnesium but not calcium, have decreased in the grain of modern wheats, particularly since the introduction of short types in the 1970s (Fan *et al*. 2008; Murphy *et al*. 2008). However, the effect on selenium is less clear, with Murphy *et al*. (2008) reporting a decrease and Fan *et al*. (2007) no significant change, with differences resulting from variation in sulphur inputs. The grain accounts for a higher proportion of the total biomass in these wheats, resulting in higher yields. Hence, the decreased concentrations of minerals may be partially due to ‘yield dilution’ (*i.e*. to increased starch accumulation). However, decreases in mineral concentrations are also observed under growth conditions in which the yield is not increased (Fan *et al*. 2008) suggesting that the dwarfing genes used to reduce plant height may have other effects. We have discussed strategies to increase the concentrations of iron and zinc (the two most important micronutrients which limit human health) in wheat grain in a previous article in this journal (Balk *et al*. 2019) and readers are referred to this for a detailed discussion.

The evidence for effects of modern breeding on other aspects of grain composition is generally weak. One reason for this is the lack of robust datasets from well‐designed experiments. In particular, most studies have compared small numbers of varieties with a limited range of release dates. The present article therefore focuses on this topic, highlighting the results of three studies from our own programmes and referring to other published work where relevant.

## Studies included and analysis of data

The first study formed part of *HEALTHGRAIN*, a multinational 5‐year (2005‐2010) EU programme which has been discussed previously in this journal (Shewry 2009). The ‘HEALTHGRAIN Diversity Screen’ compared the compositions of 150 wheat lines (130 winter and 20 spring type) grown in Martonvásár in Hungary in 2005. The lines were selected to represent a wide range of diversity, including geographical distribution and release dates, but with an emphasis on European varieties from the last 50 years (Ward *et al*. 2008). The wide expertise of the multinational partners allowed a range of components to be determined, and it remains the largest study of wheat diversity published to date (Ward *et al*. 2008). The concentrations of some components have been reported previously in relation to the release dates of the varieties (Shewry *et al*. 2011a) and relationships with further components are reported here. The 150 *HEALTHGRAIN* wheats included 5 breeding lines which are not included in the analysis here because they were not grown commercially. They also include two landraces (Chinese Spring, Nap Hal) which do not have release dates and Red Fife which was released in 1842. These three varieties are presented with a release date of 1900 for ease of viewing of the figures. Finally, more detailed fibre analyses are presented on a subset of 123 winter varieties.

However, the *HEALTHGRAIN* study had three weaknesses. Firstly, the lines were grown on a single site for 1 year without replication, and it was therefore not possible to partition the variation between the effects of genotype, environment and genotype by environment interactions. Secondly, many of the lines were grown outside their area of adaptation, which could have impacts on grain composition. Thirdly, most of the analyses were carried out on wholegrain, whereas white bread remains the dominant wheat‐based food in many countries.

In order to address these three issues, we have since analysed a further set of samples (Lovegrove *et al*. 2020). This comprises 39 lines grown in three replicate plots in the UK over 3 years. The lines were selected to represent a range of release dates, from 1790 to 2012, and for their adaptation to the UK: all had been grown commercially in the UK and, with four exceptions, bred by UK‐based breeders. Furthermore, white flour was prepared and analysed, to provide data relevant to the consumption of white bread. We will refer to this set of samples as the ‘UK Heritage Wheats’.

Thirdly, in order to specifically address the question of effects on protein content and composition, we present data from a third set of material comprising 20 Austrian wheats dating from between 1850 and 2016 which were grown in duplicate plots for two years (Call *et al*. 2020). We will refer to this set of samples as the ‘Austrian Heritage Wheats’.

For ease of comparison, the datasets are displayed as scatter plots, comparing the dates of registration of the varieties (called release dates in the text) (x axis) with the concentrations of components (y axis), with lines fitted where appropriate.

## Protein content

**Figure 1:**
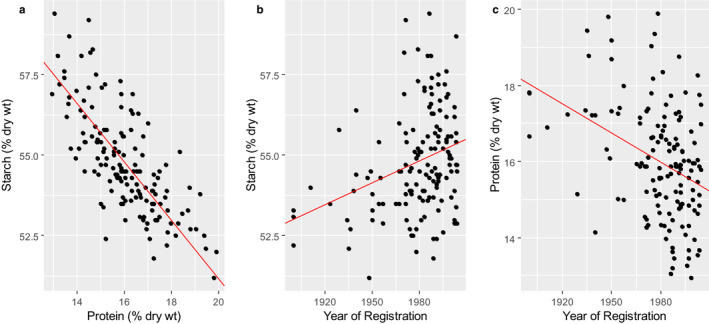
The relationships between the contents of starch (determined by NIR), protein (determined as Kjeldahl N x 5.7) and variety registration dates in wholegrain samples from the HEALTHGRAIN Diversity survey. Orthogonal regression was used to fit the line drawn in Panel (a) in order to take account of experimental errors in both starch and protein measurements. The fitted line explained 87% of the variation. Simple linear regression was used to fit the lines drawn in Panels (b) and (c) allowing experimental errors only in the y axis. The fitted lines explained 8% and 13% (adjusted *r* ^2^ values) of the variation, respectively. Despite the small *r* ^2^, both regression lines explained a statistically significant amount of the variation (*P* = 0.00018 and *P* < 0.0001, respectively). Data are from Rakszegi *et al*. (2008).

It is frequently suggested that the emphasis on breadmaking quality has resulted in modern wheats having higher contents of protein than older types. Comparisons of modern and old types grown under the same conditions in Europe do not support this. The primary target of wheat breeders over the past century has been increased yield. Higher yield results mainly from increased accumulation of starch, which dilutes other grain components including protein. This is clearly illustrated by the *HEALTHGRAIN* Diversity samples which show a clear negative correlation between the contents of starch and protein in the samples (Fig. 1a), while Fig. 1 parts b and c show increased starch and decreased protein over time, respectively.

These results are supported by the analysis of historical datasets from the US where levels of fertiliser use remain low. Kasarda (2013) analysed the available datasets for the major wheat‐growing areas in the US (Kansas and the Northern Plains) and found no evidence of increased grain protein during the 20^th^ century. However, two studies have shown small increases in the protein content of wheat grown in Canada where the yields are lower (about half) than those from the high input systems used in Western Europe (Hucl *et al*. 2015; Iqbal *et al*. 2016).

## Proteins which cause adverse reactions

The last decade has seen an increasing number of consumers adopting gluten‐free or low gluten diets, due to concerns that wheat, and gluten in particular, has detrimental effects on health. This trend is, to some extent, a lifestyle choice, driven by the popular press and social media. However, there are genuine concerns relating to the roles of gluten (or wheat) in three types of adverse response: allergy, intolerance (principally coeliac disease) and a less well‐defined syndrome referred to as non‐coeliac gluten sensitivity (NCGS) (Sapone *et al*. 2012).

The aetiology of true (IgE‐mediated) allergy to wheat consumption is well understood, and the prevalence is low (about 0.2%) (Zuidmeer *et al*. 2008). It will therefore not be discussed further here.

Coeliac disease affects about 1% of the population in the UK and Western Europe (reviewed by Shewry & Hey 2016). The aetiology of coeliac disease is again well understood. It is triggered by the consumption of wheat gluten and related proteins from barley and rye, and over 30 short amino acid sequences which trigger coeliac disease (epitopes) have been identified (Sollid *et al*. 2012). Gluten proteins are divided into two broad groups, gliadins and glutenins, with each group comprising multiple components. Analyses of wheat gluten protein sequences (*e.g*. Bromilow *et al*. 2017) show that gliadins and glutenins vary widely in their contents of coeliac epitopes, with gliadins, and particularly α‐gliadins, being richer in epitopes than glutenins (Gilissen *et al*. 2014; Shewry & Tatham 2016). Hence, increases in the proportions of gliadins could result in increases in coeliac‐toxic epitopes.

**Figure 2:**
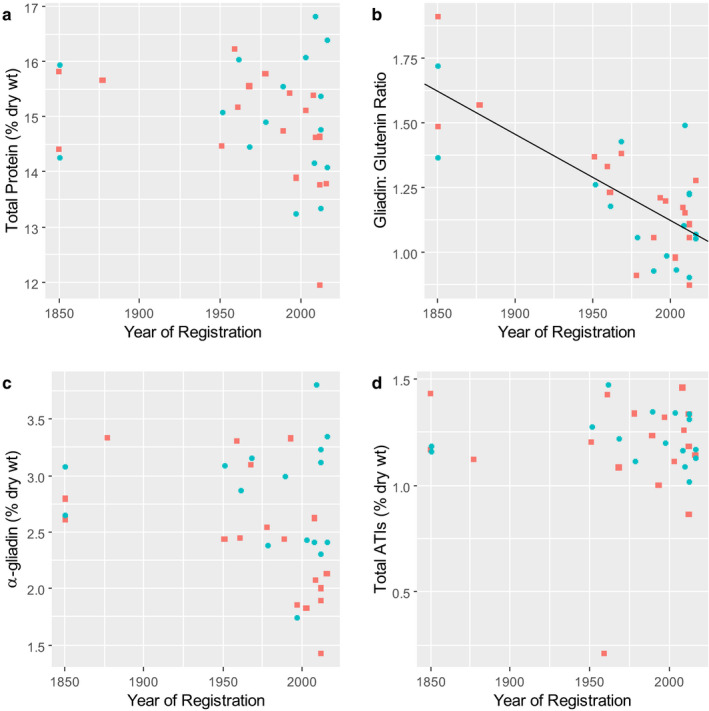
Concentration of total protein (determined as Dumas N x 5.7) (a), the ratio of gliadins to glutenins (b) and the concentrations of α‐gliadin (c) and amylase trypsin inhibitors (ATIs) (d), in wholegrain samples from the Austrian Heritage Wheats grown in 2017 (red squares) and 2018 (blue circles). Simple linear regression lines were fitted to each variable and are included in the figure where they explained a significant amount of the variation (*i.e*. where *P* > 0.05). The line shown in Panel C explains 54% of the variation (adjusted *r* ^2^) and is statistically significant *P* < 0.0001. Data from Call *et al*. (2020).

Analysis of the Austrian Heritage Wheats showed no statistically significant relationship between total protein content and release date (Fig. 2a). Analysis of gluten protein fractions showed significantly increased proportions of glutenin and decreased proportions of gliadins, resulting in a decrease in the gliadin:glutenin ratio (Fig. 2b). However, no significant effects on the proportions of the α‐gliadins were observed (Fig. 2c). These changes in gluten protein composition may reflect selection by breeders for high dough strength (which is determined by the glutenin proteins). Hence, analysis of this set of samples indicates that the relative abundance of coeliac disease epitopes is more likely to have decreased than increased in modern varieties.

Other workers have used monoclonal antibodies to directly determine the abundances of coeliac epitopes in old and modern wheats. van den Broeck *et al*. (2010) used immunoblotting to determine the relative abundances in 36 modern wheat varieties and 50 traditional wheats (called landraces) of sequences reacting with two monoclonal antibodies which recognise the minor Gli‐A20 coeliac disease epitope and the major Glia‐A9 coeliac disease epitope, respectively. Modern varieties tended to show higher reactivity with the Glia‐A9 antibody and lower reaction with the Glia‐A20 antibody; lines showing high and low reactions with both antibodies were, however, present in both sets of wheats. More recently, Ribeiro *et al*. (2016) found no relationship between coeliac toxicity and the age of the genotype, by screening 53 modern varieties and 19 landraces with the commercially available R5 monoclonal antibody which recognises a number of widely distributed coeliac‐toxic sequences. Therefore, there is no evidence that modern types of wheat are more active in triggering coeliac disease than older types.

The third type of adverse reaction to wheat, NCGS, is less well defined in terms of its prevalence, symptoms, aetiology and causative agent(s) (Sapone *et al*. 2012). In fact, even the relationship with gluten has not been established and it is perhaps more properly called non‐coeliac wheat sensitivity (NCWS). The most likely triggers for NCWS are a group of proteins known as ATIs (amylase trypsin inhibitors). These are the major group of soluble proteins in wheat, accounting for about 3.5‐4% of the total grain protein (Geisslitz *et al*. 2018). They have molecular weights of between 12 and 16 kD and comprise about 15 distinct subunits, some of which also occur in multiple forms. Most are inhibitors of α‐amylases from insect pests, and they are generally considered to contribute to plant protection. The contents of ATIs varied widely in the Austrian Heritage Wheats, with no statistically significant relationship to the age of the variety (Fig. 2d). Hence, the impact of ATIs on NCWS should not differ between old and recent varieties.

## Dietary fibre

Wheat is an important source of fibre in the Western diet, with bread alone providing between 17% and 21% (depending on age group) of the daily intake in the UK (Lockyer & Spiro 2020). Wheat fibre is concentrated in the bran layers, and wholemeal flour has a higher fibre content than white flour.

The contents of individual dietary fibre components in wholemeal flours of 129 of the winter wheat varieties in the *HEATHGRAIN* sample set were reported by Andersson *et al*. (2013) using the Uppsala method (Theander *et al*. 1995). Total dietary fibre ranged from 11.5‐15.5% dry wt. and arabinoxylan (the major component) from 5.53 to 7.42% dry wt. Other components were cellulose (1.67‐3.05% dry wt.), Klason lignin (0.74‐2.03% dry wt.), fructans (0.84‐1.85%) and β‐glucan (0.51‐0.96%), from previous analyses of the same samples by Gebruers *et al*. (2008). Two other components which contribute to dietary fibre, resistant starch and arabinogalactan peptide, were not measured and are discussed below in relation to white flour.

**Figure 3:**
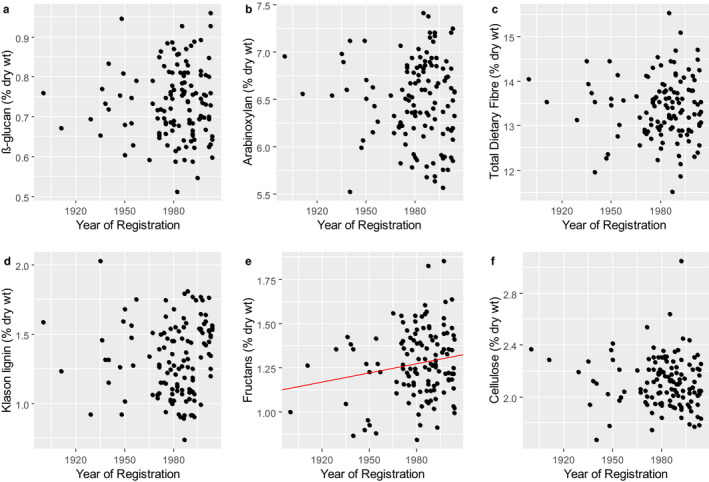
The contents of dietary fibre components in wholegrain samples of 123 winter wheats from the HEALTHGRAIN Diversity trial. Total dietary fibre is determined by the Uppsala method with the addition of fructans. Simple linear regression lines are only shown where they are found to be significant. For fructans, this was *P* = 0.051 explaining 2% of the variation according to the adjusted *r* ^2^. Data from Andersson *et al*. (2013) and Gebruers *et al*. (2008).

The registration dates of 123 of the samples were known and are plotted against the concentration of the DF components in Fig. 3. A statistically significant increase of fructan content with registration date was observed (Fig. 3e) though this only accounted for 2% of the observed variation. Hence, it can be concluded that there was little or no relationship between the fibre content and age of these cultivars.

The concentration of dietary fibre is lower in white flour than in wholemeal. The major component is again arabinoxylan (up to about 3% dry wt.) with lower concentrations of β‐glucan (about 0.5% dry wt.), fructans (about 1.5% dry wt.) and arabinogalactan peptide (up to 0.4% dry wt.) (as discussed by Hazard *et al*. 2020). Cellulose and Klason lignin are not present in white flour as they occur only in the outer layers of the grain. In addition to the fibre components discussed above, both wholegrain and white flour also contain resistant starch. This may account for up to 1% of total starch (about 0.8% dry wt. of white flour). Hence, the total content of dietary fibre in white flour ranges up to about 5% dry wt.

**Figure 4:**
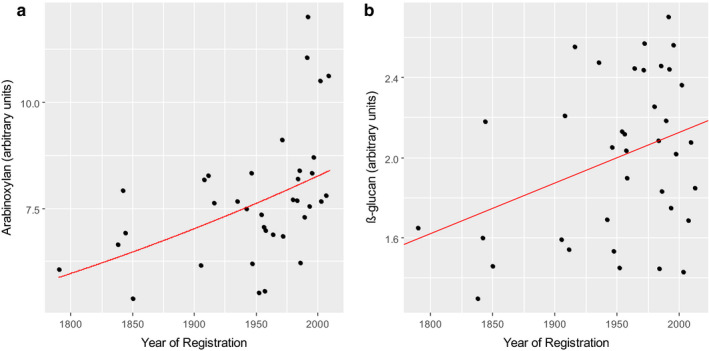
The concentrations of arabinoxylan (a) and β‐glucan (b) in white flour of the UK Heritage Wheats. Data are expressed in units determined by HPLC analysis of oligosaccharides released by enzyme digestion. Hence, the analyses are comparative between samples but do not provide precise concentration. Data are means of samples from three replicate plots grown for each of 3 years. Lines drawn are from simple linear regression and for arabinoxylan, back‐transformed from the line originally fitted on the log scale to ensure homogeneity of variance. The lines shown are statistically significant (*P* = 0.002, *P* = 0.032) explaining 21% (a) and 10% (b) of the variation. Data from Lovegrove *et al*. (2020).

Statistically significant increases in the concentrations of both arabinoxylan and β‐glucan in white flour are observed with year of registration for the UK Heritage samples (Fig. 4), explaining 21% and 10% of the variation in the datasets, respectively.

A smaller study of eight modern and seven older durum wheat varieties adapted to and grown in Italy showed no differences in the content of arabinoxylan and β‐glucan in wholemeal or refined flour (called semolina for durum wheat) (De Santis *et al*. 2018).

## Amino acids, sugars and betaine

Wheat grain and flour contain a range of soluble metabolites, including amino acids and sugars, which are readily quantified by high throughput metabolomic screens.

**Figure 5:**
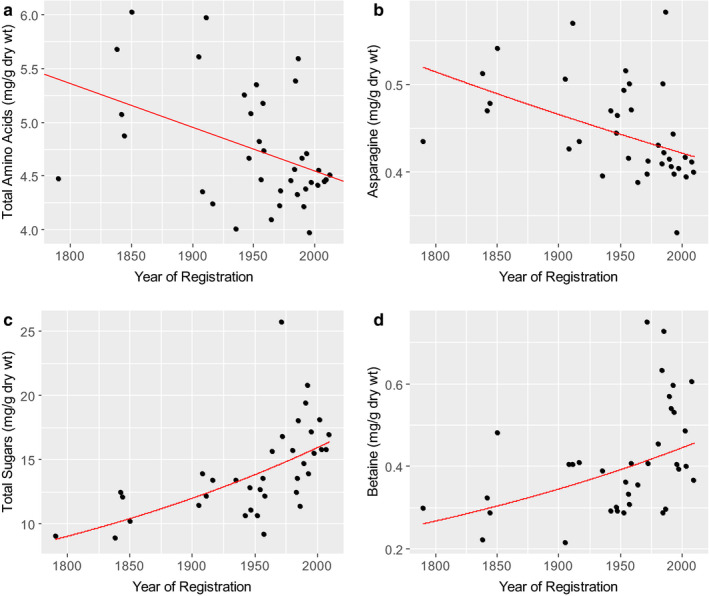
The concentrations of soluble metabolites in white flour samples from the UK Heritage lines. Data are means of samples from three replicate plots grown for each of 3 years. Lines are from simple linear regression. In the case of asparagine, total sugars and betaine, the lines shown are the back‐transformed line originally fitted on the log scale to ensure homogeneity of variance. Lines are shown when the estimated trend was statistically significant (*P* = 0.010, *P* = 0.0045, *P* < 0.0001 and *P* = 0.0037, for total amino acids, asparagine, total sugars and betaine, respectively) explaining 15%, 19%, 41% and 19% of the variation according to the adjusted *r* ^2^, respectively. Data from Lovegrove *et al*. (2020).

^1^H NMR spectroscopy of white flours (Shewry *et al*. 2017) from the UK Heritage Wheats quantified 10 individual amino acids. A clear decrease in the total concentrations of these amino acids was observed (Fig. 5a), with similar decreases in the concentrations of most individual components including asparagine (Fig. 5b) (Lovegrove *et al*. 2020). Asparagine is a precursor of acrylamide, a neurotoxin and potential carcinogen which is formed by Maillard reactions with reducing sugars during food processing, and the concentration of asparagine is usually the limiting factor for acrylamide formation in cereal products (Curtis & Halford 2016).

Sugars determined comprise monosaccharides (glucose, fructose, arabinose, galactose), disaccharides (maltose, sucrose) and the trisaccharide raffinose. The total concentrations of these components have increased significantly in the more recent varieties, particularly those introduced after 1950 (Fig. 5c). The concentrations of the individual sugars also increased, except for arabinose and galactose (Lovegrove *et al*. 2020).

It is not known why the concentrations of some individual metabolites have increased or decreased, but it is possible that the decreased concentration of total amino acids is associated with the decrease in protein, and the increases in concentrations of sugars with the increase in starch (see Fig. 1).

Metabolite profiling by ^1^H NMR spectroscopy also quantified the concentrations of choline and betaine (which is more correctly called glycine betaine). These biosynthetically related components act as ‘methyl donors’ in humans, being able to donate methyl groups for the conversion of homocysteine to methionine in the homocysteine cycle, and hence reduce the risk of cardiovascular disease (Ueland *et al*. 2005; Chiuve *et al*. 2007). Wheat is one of the richest known sources of betaine in the diet (Zeisel *et al*. 2003). Betaine is generally present at about10 times the concentration of choline in wheat grain (Corol *et al*. 2012) with both betaine and choline being concentrated in the bran (Zeisel *et al*. 2003).

Analysis of white flours of the UK Heritage Wheats showed significantly higher concentrations of betaine in the varieties released from 1980, compared with the older varieties (Fig. 5d), with no significant differences in the concentration of choline (Lovegrove *et al*. 2020). By contrast, no relationship was found between betaine content and release date in the *HEALTHGRAIN* lines (not shown).

## Phytochemicals and vitamins

Cereals are rich sources of phytochemicals, most of which fall into two major classes: phenolics and terpenoids. Individual components may differ in their distributions between grain tissues, as discussed by Piironen *et al*. (2009), but all are more abundant in wholemeal flour than in white flour. Hence, most analyses, including those discussed below, have been carried out on wholemeal rather than white flour.

### Phenolics

Phenolics contain at least one aromatic ring bearing at least one hydroxyl group. They are the most abundant phytochemicals in wheat grain, with phenolic acids being the major class. Phenolic acids occur in three forms in the wheat grain: as free compounds, as soluble conjugates bound to sugars and other low molecular weight components and as bound forms which are linked to arabinoxylan in the cell wall by ester bonds. The concentrations of phenolic acids vary widely between wheat samples, but bound forms generally account for about 80% of the total, with the major individual component being bound ferulic acid (Li *et al*. 2008).

Phenolics from plant‐based foods have been shown to improve vascular function and hence reduce the risk of cardiovascular disease (Vauzour *et al*. 2010), and similar activity has been demonstrated for ferulic acid released from arabinoxylan in wheat bread (Turner *et al*. 2020). Minor phenolic components in wheat include lignans which are derived from the combination of two phenylpropanoid (C_6_‐C_3_) units and alkylresorcinols which are phenolic lipids. Lignans act as phytoestrogens while the restriction of alkylresorcinols to the testa layer of the grain has led to their use as biomarkers to monitor the consumption of wholegrain (Piironen 2009).

**Figure 6:**
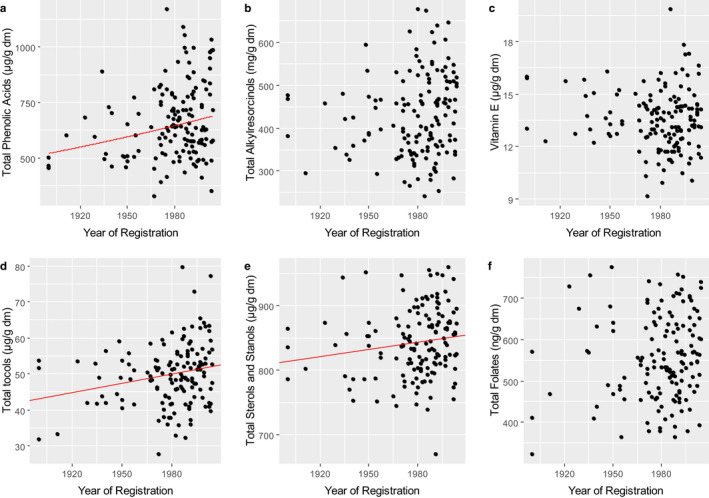
Contents of phytochemicals, including vitamin E (α‐tocopherol) (D) and vitamin B9 (folate) (F) in wholegrain samples from the HEALTHGRAIN Diversity trial. Lines are from simple linear regression, where the trend was statistically significant. In the case of total phenolic acids, the line shown is the back‐transformed line originally fitted on the log scale to ensure homogeneity of variance. Lines are shown where the estimated trend was statistically significant (*P* = 0.004, *P* = 0.008 and *P* = 0.068, for total phenolic acids, total tocols and total stanols and sterols, respectively) explaining 5%, 4% and 2% of the variation according to the adjusted *r* ^2^, respectively. Data from Li *et al*. (2008), Lampi *et al*. (2008), Nurmi *et al*. (2008) and Piironen *et al*. (2008).

Analysis of wholegrain samples of the *HEALTHGRAIN* wheats showed a statistically significant increase in the concentration of total phenolic acids with release date (Fig. 6a) but not of total alkylresorcinols (Fig. 6b). However, release date only accounted for 5% of the variation in the concentration of total phenolic acids. Similarly, comparisons of small numbers of ‘old and recent’ varieties adapted to and grown in Italy showed no difference in the total concentrations of phenolic compounds in durum or bread wheats, although the composition was more diverse in the older varieties (Heimler *et al*. 2010; Dinelli *et al*. 2011). By contrast, Dinelli *et al*. (2007) showed higher mean contents of lignans, by about 2‐fold, in six old bread wheat varieties than in four modern varieties.

### Terpenoids

Terpenoids are based on 5‐carbon isoprene units which are assembled to form larger structures and subject to a range of modifications, including cyclisation. Terpenoids in wheat include sterols, tocols and carotenoids (Piironen 2009).

Sterols comprise a tetracyclic cyclopenta[α]phenanthrene ring with a hydroxyl group at the C3 position and a flexible side chain at the C17 carbon position. Cereals contain significant amounts of saturated sterols, which are called stanols, and a substantial proportion of the sterols and stanols present in wheat are modified, either esterified to a fatty acid or phenolic acid to form sterol esters, or β‐linked to a carbohydrate to form a sterol glycoside, with the latter also sometimes being acylated. Plant sterols and stanols have well‐established health benefits, in the maintenance of normal blood cholesterol concentrations (Kritchevsky & Chen, 2005; EFSA NDA Panel 2010).

The total concentrations of sterols (including stanols) in wholemeal flours of the *HEALTHGRAIN* lines ranged from 670‐959 μg/g, with a mean of 844 μg/g (Nurmi *et al*. 2008). There was a marginally statistically significant (*P* = 0.068) correlation between the concentration of total sterols plus stanols in the samples and the release date. However, the date of registration only accounted for 2% of the variation in the dataset (Fig. 6e).

### Tocols

Tocols comprise a chromanol ring with a C16 phytol side chain, which can be either saturated (tocopherols) or unsaturated (tocotrienols). Tocopherols and tocotrienols each exist in four forms in wheat, which differ in the number and positions of methyl groups on the chromanol ring and are called α, β, γ and δ. Although the name ‘Vitamin E’ has been applied to all tocols, they differ in their activity with α‐tocopherol being the most active form (Bramley *et al*. 2000). Currently, only α‐tocopherol is considered to possess vitamin E activity (EFSA NDA Panel 2015).

The total concentration of tocols in the *HEALTHGRAIN* lines ranged from 27.6 to 79.7 μg/g (mean 49.8 μg/g) and the concentration of α‐tocopherol from 9.1 to 19.9 μg/g (Lampi *et al*. 2008). A statistically significant correlation between the concentration of total tocols and the release dates of the varieties was observed, though this only accounted for 4% of the variation in the dataset (Fig. 6d). No correlation was observed between the concentration of α‐tocopherol (Vitamin E) and release date (Fig. 6c). Hussein *et al*. (2012) similarly reported that there were no differences in the contents and compositions of tocols between a smaller sample set of landraces (8 genotypes), old cultivars (13) and modern cultivars (2) of bread wheat.

### B vitamins

The B vitamin complex comprises eight water‐soluble components which often occur together in the same foods. Although they were initially considered to be a single compound, the individual vitamins are not related. Cereals, including wheat, are important sources of B vitamins, providing about a third of the total daily intake of thiamine (B1), 27% of the intake of niacin (B3) and 33% of the intake of folate (B9) by adults in the UK (Lockyer & Spiro 2020).

Wide variation has been reported in the contents of B vitamins in wheat (Piironen 2009; Shewry *et al*. 2011b; Shewry & Hey 2015). Six forms of folate, called vitamers, were determined in wheat and their total concentrations in wholemeal flours of the *HEALTHGRAIN* lines ranged from 0.32 to 0.77 μg/g (mean 0.56 μg/g). The proportions of the individual vitamers varied between lines but contributed on average from 6 to 41% of the total (Piironen *et al*. 2008). No relationship between the total concentration of folate and the age of the varieties was observed (Fig. 6f).

## Discussion

It is clear from the studies discussed above that intensive wheat breeding has resulted in increased accumulation of starch, which is generally associated with a decrease in the concentration of protein. Analysis of the Austrian Heritage lines also indicates that there have not been increases in proteins known to trigger adverse reactions. Other effects of breeding on grain composition are less clear, and the studies discussed in detail here demonstrate the challenges.

One major challenge is that grain composition is strongly affected by the environment (Shewry *et al*. 2010). Hence, it is essential to compare material grown in replicated multi‐environment field trials. Furthermore, the varieties compared should be adapted to the area of growth, to avoid the effects of environmental stress. The *HEALTHGRAIN* study clearly did not fulfil these criteria, and it is not surprising that few correlations were observed, and, with the exception of starch and protein, these were marginal in significance (accounting for between 2% and 5% of the variation observed in the analyses). Nevertheless, the analyses are of interest in that they show no major changes in composition.

By contrast, the UK Heritage Wheat samples were from replicated multi‐site trials with an emphasis on flour composition. Statistical analyses of these samples showed positive correlations of release date with the contents of arabinoxylan fibre (accounting for 21% of the total variation), total sugars (41%) and betaine (19%), and negative correlations with total amino acids (15%) and individual amino acids including asparagine (Lovegrove *et al*. 2020). These changes have clear implications for human health.

Wheat is the most important single source of dietary fibre in many diets, including the UK and Western Europe, and the increased content of arabinoxylan (the major fibre component) in white flour is certainly desirable. The decreased concentration of asparagine in modern wheats is also desirable as it reduces the potential for the formation of acrylamide during processing.

By contrast, the increases in fermentable monosaccharides, disaccharides and oligosaccharides (sucrose, mannitol, fructans) may be of concern to consumers suffering from irritable bowel syndrome (IBS), as these form part of the FODMAP fraction (fermentable oligosaccharides, disaccharides, monosaccharides and polyols) that exacerbate IBS symptoms (Gibson & Shepherd 2010). However, wheat is already recognised as a major source of FODMAPs in the diet (Biesiekierski *et al*. 2011; Whelan *et al*. 2011) and excluded by many IBS patients.

To conclude, the analyses discussed provide no evidence that modern types of wheat have lower quality for human nutrition and health, with the exception of decreased levels of some minerals (including iron, zinc and magnesium) which are discussed elsewhere. In fact, there is evidence that they may be superior in some respects, particularly in fibre content of white flour. However, the analyses also show the challenges facing researchers and the need for more datasets from well‐designed field trials.

## Conflicts of interest

The authors have no conflicts of interest to disclose.
